# Pediatric safety: review of the susceptibility of children with disabilities to injuries involving movement related events

**DOI:** 10.1186/s40621-019-0189-8

**Published:** 2019-04-08

**Authors:** Abbey Fraser, Dao Doan, Mary Lundy, Grant Bevill, Juan Aceros

**Affiliations:** 1 College of Computing, Engineering & Construction, University of North Florida, 1 UNF Dr, Jacksonville, FL 32224 USA; 2 Brooks College of Health, University of North Florida, 1 UNF Dr, Jacksonville, FL 32224 USA

**Keywords:** Toy-related injuries, Ride-on toy, Pediatric safety, Disabilities, Collision, Children

## Abstract

**Background:**

Toy-related injuries have increased significantly in the past decade, in particular those related to ride-on toys. This increase has been attributed to movement related events such as falls and inertial impacts. Furthermore, children with disabilities have been reported to be at a greater risk of being injured, and are therefore more susceptible to toy-related injuries. Although, efforts are being made to modify ride-on toys as a method for increasing quality of life in children with disabilities, there are very limited pediatric safety studies regarding children with disabilities and modified ride-on toys.

**Methods:**

This manuscript presents a systematic review of literature summarizing the current state of toy-related injuries including children with and without disabilities. Children exposed to inertial impacts in motor vehicle crashes have also been reviewed to present current pediatric safety testing methodologies and injury tolerance thresholds. Out of 2608 articles, 10 studies were included regarding current trends in toy-related injuries and safety testing methodologies.

**Conclusions:**

From this study, a gap in the literature was discovered concerning the susceptibility of children with disabilities to toy-related injuries, specifically in relation to ride-on toys and the repercussion surrounding such injuries. It is theorized that such lack of data is due to the difficulty and costs associated with experimental validation. Hence, it is recommended that computer simulations be used to provide preliminary data analysis.

## Background

Pediatric safety had garnered more attention as of late in the scientific community (e.g. car seat safety, modified ride on toys for children with disabilities, etc.). Despite this recent interest, limitations exist in the knowledge of pediatric safety testing and tolerance thresholds due to a limited amount of test data. Children are unable to volunteer as test subjects and child cadavers are not readily available for research. Often, research is done using anthropomorphic test dummies that model the average child. This presents a problem however because it does not account for children with disabilities. Moreover, in recent years, children with disabilities have seen an increase in opportunities for transportation due to power mobility technologies and modified ride-on toys. These modified vehicles provide children with disabilities the chance to play and move in their environment.

This is a significant advancement for children, because toddlers and preschoolers require independent exploration of their environment for their brain cells and neural connections to develop properly (Thompson 2001). Around six months of age children will seek to move toward items that capture their interest and engage in independent, self-directed exploratory play (Thompson 2001). Toys help children participate in these activities and develop the necessary cognitive, social, and motor skills to manage a fulfilling life (Ginsburg 2007). This is true, and especially important, for children whose disabilities often keep them from engaging in normal play. As part of these independent, exploratory play activities, accidents are bound to happen and expected as part of their development.

Pediatric safety research and regulation concentrates on protecting children from hazards such as toy-related injuries. It has been reported that the annual injury rate of toy-related injuries in children has increased from 1990 to 2011 by 39.9%. This has been highly correlated to injuries caused by ride-on toys, as they account for 34.9% of all injuries (Gaw, Abraham, Gaw, Chounthirath, & Smith 2015). Most injuries caused by ride-on toys are due to falls and inertial impacts. Furthermore, the number and rate of injuries have been reported to peak at 2 years of age -an age when children are still beginning to learn about their environment through movement and play-.

In recent years, researchers have engaged in the development of adaptive technology in the form of modified ride-on toys to provide children with disabilities independent self-directed mobility (Logan, S. W., Feldner, H. A., Galloway, J. C., & Huang, H. 2016). It is noted that for this manuscript, a ride-on toy was defined as any rideable toy used for the purpose of play. Also, limited work has been done in studying the safety of such devices, and how modifications can worsen or improve the safety of a device in relation to a disability. Most studies have been conducted reviewing toy-related injuries in children without disabilities. One study comprehensively investigated toy-related injuries from a nationally representative data set collected from 1990 to 2011. This was the first time such a study was conducted. Mechanism of injuries were defined and separated into categories. Children were divided by age as well: 5 years of age or younger, and age 5 to 17. Most injuries were found to be caused by ride-on toys and this number increased by 73.7% from 1990 to 2011. It was hypothesized this is due to the increased popularity and accessibility of ride-on toys. These injuries spiked in 2000 and 2001. However, due to increased safety regulations, a noticeable decline in ride-on toy related injuries was observed. This displays the importance and impact of safer design and increased regulation.

A few other studies have investigated the susceptibility of children with disabilities to injury. One pooled data from the years 1997–2005 from the National Health Interview Survey, which is a multipurpose health survey completed annually by the United States Census Bureau, for the National Center for Health Statistics. To compare prevalence of injury between children with and without disabilities, a child with disabilities was matched to a healthy child of the same gender and age. It was determined that socioeconomic variables were insignificant. It was also found that children with disabilities experience a higher rate of injury (3.8% vs 2.5%; *P* < .01). It was found that the risk of injury varied by the type of disability such that the more severe the disability, the higher the rate of injury.

Another study utilized the China Disabled Persons’ Federation (CDPF) to conduct a study on all children with disabilities ages 1–14 (H. Zhu et al. 2014). The CDPF maintains a registry database that monitors the number of persons with disabilities and tracks the medical and rehabilitation services provided by the government. Each child with a disability was matched with a healthy child of same gender, age, and living in the same neighborhood for the study. Disabilities were categorized as vision, hearing, speech, physical disabilities, intellectual disabilities, and mental health disorders. Children were also organized by four levels of disabilities, defined as level four being most severe and nonfunctional, level three being less severe and minimal functionality, level two being semi-functional and level one being the mildest degree of disability with the best functionality. Sociodemographic variables were taken into consideration, including: gender and age of the child, parent’s education, family income, single-parent family status, time of being supervised by an adult each day, and total number of family members (H. Zhu et al. 2014).

Injuries were assessed when an injured child sought medical care at a hospital or community clinic. The rate of injury among healthy children was found to be 4.4%, but increased to 9.6% for children with a single disability, and 11.2% for children with multiple disabilities. The level of disability that was injured the most was level 2 (11.5%), followed by level 3 (10.4%), level 4 (10.3%), and level 1 (8.1%) (Klevberg, Elkjaer, Jahnsen, Elvrum, Zucknick, Ostensjo, & Krumlinde-Sundholm 2018). Emerging evidence indicates that individuals with disabilities face a significantly higher risk of injury than those without disability.

## Systematic literature search and data extraction

Two independent researchers performed a systematic literature search to identify all relevant studies pertaining to pediatric safety and toy-related injuries for children with and without disabilities. Due to the limited number of studies on toy safety for children with disabilities, the search was divided into two sections. Section 1 focused on toy-related injuries. Section 2 focused on inertial impact and injury risk in vehicular collisions. This information is relevant due to its relation to inertial impacts caused by ride-on toys.

The following search terms were used: pediatric OR children OR pediatric OR child OR infant AND injury OR injuries OR accident OR trauma AND disability OR disabilities OR disabled OR handicap for the first section. Search terms safety AND inertia impact OR crash or “car crash” AND ATDs OR cadavers OR “computer simulations” AND “low-speed” AND scaling AND scaling techniques AND crash analysis were added to the first search terms for the second section.

**Fig. 1:**
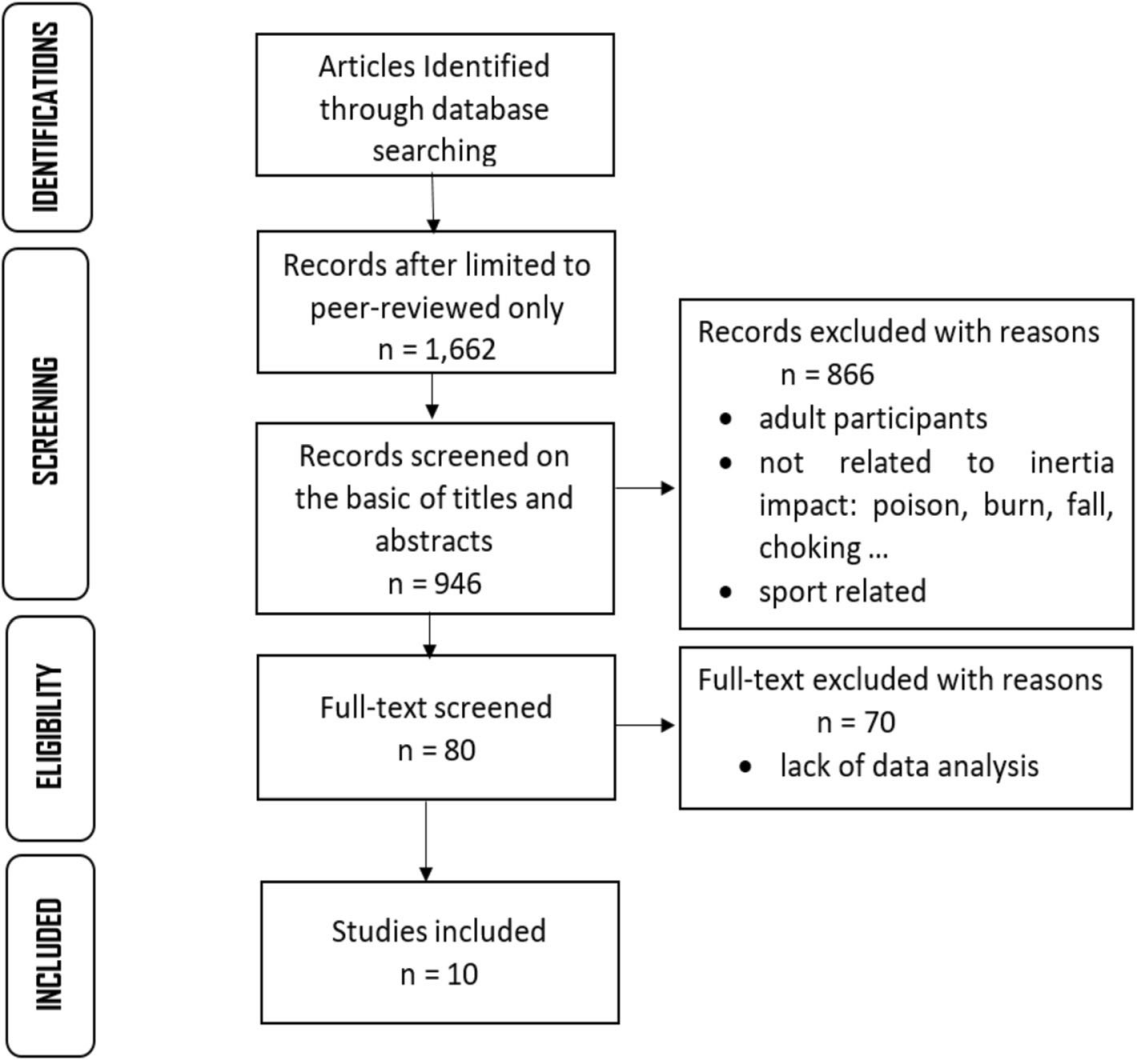
Search process and identification of relevant studies

All titles that were relevant to the criteria went through a subsequent screening based on their abstract, and full text articles were reviewed once they were determined appropriate for this study. Studies were excluded when: high velocity impact injuries were surveyed, there was insufficient data concerning children with disabilities, adult participants were included. Fig. 1 describes the process of search and screening.

## Results: Methodology employed in the studies

### Pediatric safety testing

#### Safety testing by anthropometric test devices (ATDs)

Two studies investigated child safety in motor vehicle impacts by conducting a series of sled tests with 6 and 10-year-old anthropometric test devices (ATDs) using 48–50 km/hr. frontal crash pulse. One study investigated injury risk of a pediatric occupant with a disability sitting in a wheelchair while being transported (Ha & Bertocci 2007). The other studied the possibilities of better protection when children were not using booster seats. Methods of increasing protection that were studied include various cushion lengths and varying lap belt geometry (Rosenbaum & Rosenbloom 2012). Table 1 shows an overview of the studies.

**Table 1:** Overview about the studies’ objectives, impact conditions, methodologies of reviewed pediatric safety studies

Author	Objective & Population	Impact Conditions	Methodology	Results & Recommendations
Gaw,C. Abraham, V. M., Gaw, C. E., Chounthirath, T., & Smith, G. A. (Park & Yoo, 2009)	Comprehensively investigate toy-related injuries among children	N/A	Patients were separated into 2 age categories, younger than 5 years, and 5 to 17 years of age. Mechanism of injury was divided into categories such as falls, collisions and foreign body involvement.	Ride-on toys were 3.19 times more likely to be associated with a fracture or dislocation compared with other toy products. Patients younger than 5 years were more likely to injure their head or neck and face than patients aged 5 to 17. 34.9% of toy-related injuries were associated with ride-on toys.
Sinclair, S. A., & Xiang, H. (Blankenburg et al., 2018)	Verify reports from many researchers that report that disabled children are at a higher risk of injury than non-disabled children. The epidemiology of injury among children with disabilities hasn’t been adequately studied.	N/A	Data was pooled from the 1997–2005 National Health Interview Survey (NHIS) The prevalence of injuries in children who had a single disability were compared to children without a disability by gender, age, parent’s education, poverty status, and family size. An injury episode was defined as a traumatic event in which the person was injured 1 or more times from an external cause.	It was found that the risk of injury was significantly higher among children with a single disability than among non-disabled children. (3.8%; 95% CI = 3.4, 4.1 vs. 2.5%; 95% CI = 2.5, 2.6, respectively; *P* < 0.001). However, the risk of injury differs by type of disability. The most frequent causes of injury episodes for both test groups were falls. The disability with the greatest probability of injury was children who had a bone, joint, or muscle problem.
Zhu, H., Xiang, H., Xia, X., Yang, X., Li, D., Stallones, L., & Du, Y. Â (AlemdaroÄŸlu, 2017)	Children with disabilities may have a reduced ability to handle environmental hazards because of physical limitations, impairments in mental processing, or in their ability to adjust to their environment.	N/A	The China Disabled Persons’ Federation was utilized to survey all registered, disabled children ages 1–14 years. For every disabled child, a non-disabled child living in the same neighborhood and with the same gender and age was matched. Disabilities were organized into categories of vision, hearing, speech, physical, and mental health disorders. Children with multiple disabilities were also taken into consideration. A scale of four varying levels of disabilities was used. Socio demographic variables were also considered.	Rates of injuries among children with a single disability (9.6%) and multiple disabilities (11.2%) were significantly higher than that among children without disabilities (4.4%). It was found that age of the child, children in single parent households; children whose parent’s highest education was middle school or less; children with less than 30% of time per day supervised by an adult; and children whose family income per month was less than 1000 RMB has little to no change on rate of injury. Level 2 of disability was injured the most (11.5%), followed by level 3 (10.4%), then level 4 (10.3%), and lastly level 1 (8.1%).
Ha D. (Ginsburg, 2007)	6-year-old children with disabilities 3 pediatric manual wheelchairs	20 g/48 km/h front crash pulse Wheelchair impact	Sled test using a seated Hybrid III 6-year-old ATD Head acceleration, chest acceleration, pelvis acceleration, femur forces, chest deflection, neck forces, and moment were measured.	Test results were compared with kinematic limitation and injury criteria that listed in the ANSI/RESNA WC-19, FMVSS 213 and FMVSS 208 standards.
Klinich KD (Kleinberger et al., n.d.)	6-year-old and 10-year-old healthy children Not using belt-positioning booster	Sled pulse delta velocity is 28.8 km/hr.	Sled test using a seated Hybrid III 6-year-old and 10-year-old ATD Vehicle seats Cushion length of 450 mm Cushion length of 350 mm Belt Geometry Lap belt angles tested: 23^o^ (rear), 50^o^ (mid),and 70^o^ (forward)	Compared kinematic outcomes between long and short cushion length and increasing lap belt angles.
Park D (Zhu et al., 2014)	Healthy 3-year-old children Existing three-point belt-type child seat	50 km/h front crash pulse Children car seat impact	Combined sled test and computer based simulations a Standard crash test: velocity increased to 50 km/h then suddenly decelerated Simulation: Geometric modelling: LS-DYNA, CATIA Preprocessor: FEMB, and postprocessor: LS-POST Finite elements	Compared sled test and computer simulation results to validate data collected. Developed an advanced new type of a child seat based on the results (six-point belt-type)
Isabelle S. (Sinclair & Xiang, 2008)	Compare the kinematic response of children and child anthropomorphic test devices (ATDs) during emergency braking events in different restraint configurations in a passenger vehicle 16 healthy children aged 4 to 12 Q3, Hybrid III (HIII) 3-year-old, 6-year-old, and 10-year-old ATDs	2 braking events Vehicle brakes as fast as possible to a full stop while traveling at a velocity of 70 km/h The maximum deceleration of all analyzed braking events was 1.2 g. The peak mean deceleration was 1.0 g with a standard deviation of 0.08 g and duration of 1.8 s The duration of the entire deceleration period was 2.4 s 2 sharp turns to the right in each restraint system	Child volunteer and ATDs test Short children (stature 107–123 cm) and the Q3, HIII 3-year-old, and 6-year-old were restrained on booster cushions as well as high-back booster seats. Tall children (stature 135–150 cm) and HIII 10-year-old were restrained on booster cushions or restrained by 3-point belts directly on the car seat. Restrained on the right rear seat of a modern passenger vehicle. Four small video cameras (Monacor TVCCD-30, lens focal length 3.6 mm, Monacor International, Bremen, Germany) were affixed inside the vehicle providing a front view of the child, a perpendicular lateral view, and 2 oblique views of the children volunteer. The recording rate was 12.5 frames per second. Data collected included vehicle velocity, acceleration in longitudinal and lateral directions, and brake pressure. MATLAB was used to analyze data.	40 trials were analyzed Child volunteers had greater maximum forward displacement of the head and greater head rotation compared to the ATDs. The average maximum displacement for children ranged from 165 to 210 mm and 155 to 195 mm for the forehead and ear target, respectively. Corresponding values for the ATDs were 55 to 165 mm and 50 to 160 mm. The change in head angle was greater for short children than for tall children. Shoulder belt force was within the same range for short children when restrained on booster cushions or high-back booster seats. For tall children, the shoulder belt force was greater when restrained on booster cushions compared to being restrained by seat belts directly on the car seat
Jingwen H. (Gaw et al., 2015)	Analyses of crash injury data have shown that injury risk increases when children transition from belt-positioning boosters to the vehicle seat belt alone. Investigate how to improve the restraint environment for these children. Healthy children aged 6 to 12 years old	Frontal crash test	Used a parametric child ATD MADYMO model To scale the baseline child ATD model into different body sizes, custom software was developed by combining MADYMO Scaler and a program written by Scilab V5.2.2 (Scilab Enterprises, France) An automated computer program was developed using a combination of MADYMO (TASS, The Netherlands), Scilab, and ModeFRONTIER (ESTECO, Italy) to integrate the parametric child ATD model, ATD positioning procedure, automatic belt fitting algorithm, and other crash conditions together. A 200 N force was applied to the 3 belt anchorages	The maximum head and knee excursions in this parametric study were 639 and 833 mm, respectively. Both were below the limits defined in FMVSS No. 213, in which head excursion should be less than 720 mm and knee excursion should be less than 915 mm. Lower and more rearward D-rings (upper belt anchorages), higher and more forward lap belt anchorages, and shorter, stiffer, and thinner seat cushions were associated with improved restraint performance. Children with smaller body sizes require more-forward D-rings, inboard anchors, and outboard anchor locations to avoid submarining. However, these anchorage locations increase head excursions relative to more-rearward anchorages.
Florian F. (Logan et al., 2016)	Case study: a serious accident involving two passenger cars took place in Austria in which three children seated in the rear were fatally injured in a frontal collision. The study was performed to gain a better understanding of rear occupants injury mechanisms and potential improvements to rear-seat restraint system 3 children: 5 years old, 8 years old and 10 years old	Frontal collision EBS (equivalent barrier speed) and EES (energy equivalent speed) is 62 km/h Approaching angle of 85â—¦ The approaching velocity of the VW was calculated to be 63 km/h For the Nissan, a velocity of 69 km/h was determined	An HIII (hybrid III) six-year-old dummy (hereafter HIII 6yo) was used for simulating the youngest child, aged five, seated behind the driver. The eight-year-old child, who was seated in the middle, was simulated by a TNO P10 dummy (hereafter TNO P10) For the eldest child, aged 10, an HIII 5th percentile dummy (hereafter HIII 5th) was used An HIII 50th percentile dummy was seated in the driver’s seat. An HIII 5th percentile dummy was situated on the front occupant’s seat to enable direct comparison of the restraining effect between the front and the rear compartments A crash test was used for validating a numerical model of the rear compartment, programmed with the multi-body (MB) simulation code MADYMOR. The MADYMOR model was used for a set of parametric variations	Results: The HIII 5th seated in the rear showed a considerable chest (52 mm chest deflection, 66 g chest acceleration) and head load (HIC [head injury criterion] = 1047 and acceleration exceeding during a cumulative time interval of 3 ms [cum3ms] = 96 g). The shoulder belt forces reached almost 9 kN ◦ The chest deflection in the HIII 6yo and HIII 5th only slightly exceeded the threshold values of 40 mm and 52 mm In contrast, the loads on the HIII 5th seated in the front seat were consistently lower compared with those on the rear-seated HIII: head acceleration was 25% lower, neck forces and torques were considerably lower (by 25–40%), chest deflection was 25% lower, shoulder-belt forces were 12% lower and chest acceleration was 15% lower. Furthermore, the shoulder belt in the front seat had a 50% greater pullout (100 mm) Recommendations Provision of mandatory seatbacks with side wings to protect against lateral impact. Provision of a mandatory guide for shoulder belts. Mandatory introduction of anti-rotation devices, e.g., top tether and outrigger. Definition of maximum size of not-ISOFIX seat (geometry envelope). Identification of CRS, including the weight, size and age of the child for which the specific model is designed.

Data related to head, chest, and pelvis acceleration, femur and neck forces, chest compression, chest deflection, and moment were measured during each test. The results from the wheelchair occupant study were then compared with the kinematic limitations and injury criteria of the Federal Motor Vehicle Safety Standards (FMVSS). Comparison to FMVSS 213 (Safety standard that must be met for children car seats to be sold for use. Includes requirements such as the child restraint system must pass a 30 miles per hour frontal sled test that simulates a crash, padding requirements, flammability standards, and buckle release pressure.) and FMVSS 208 (Safety standard for occupant crash protection that establishes performance requirements for passenger vehicles (Lewandowski 2006)) was used to determine the injury risk of the pediatric wheelchair occupant in a motor vehicle crash (Ha & Bertocci 2007).

The study that investigated alternative seat belt protection used head excursion, peak knee excursion, the difference between peak head and peak knee excursion, and maximum torso angle to determine whether sitting with a shorter cushion and mid or forward angle lap belt would be better for safety when children are not using booster seats.

#### Safety testing by ATDs and computer simulations

One study used the combination of sled testing and computer simulations to develop an advanced child restraint system (CRS) (Park & Yoo 2009).

A sled test was first performed using a 3-year-old ATD in an existing three-point seat belt CRS with the objective of achieving head and chest accelerations within safety limits. The crash test was designed to exert accelerations according to national standards, and increased velocity to 50 km/h and then suddenly decelerated. A dynamic simulation was then conducted using a commercial LS-DYNA® program developed by Livermore software Technology Corporation. LS-DYNA® was used for contact and collisions, and the computer-aided three-dimensional interactive application (CATIA™) program was used for geometric modeling. Once the sled test and computer simulation results was matched, a new type of child seat was developed. An optimization sequence was applied to determine the thickness of each part to decrease the weight. A new six-point CRS was then developed using LS-DYNA®. Once the final result was obtained from the computer simulation of the new design, the sled test was carried out with the developed prototype of a six-point child seat (Park & Yoo 2009). An overview is shown in Table 1.

## Results: Main findings of the studies

### Pediatric safety testing

#### Safety testing by anthropometric test devices (ATDs)

Sled tests were conducted under 48 km/h and 20 g average impact conditions on children riding in a motor vehicle while seated in a wheelchair. This study showed that a 6-year-old seated in a wheelchair may be at risk of neck injury during a frontal car crash and concluded that variations in the shoulder belt anchor point led to variance in restraint effectiveness. All tests conducted in this study exceeded the tension extension limit. All tests complied with the requirement that the wheelchair not load the ATD. None of the tests exceeded the limit which evaluates the integrity of seat surface and seat attachment hardware and none of the tests exceeded the maximum chest acceleration limit of 60 g. The results of the safety testing satisfied the head injury criterion (HIC) of 700 which measures the amount of damage to the head. Chest deflection for two iterations of the test were at the limit of 40 mm specified in regulation. The first and second sled test exceeded the peak neck tension force limit of 1490 N. No tests exceeded the independent compressive neck force limits (Ha & Bertocci 2007).

When cushion size and lap belt angle were tested, increased cushion length and bigger lap belt angles improved children safety in a motor crash. However, seat boosters still have the best child safety performance than simply increasing the cushion length and lap belt angle.

#### Safety testing by ATDs and computer simulations

While the study failed to match the sled test results to simulations results exactly, the collected data and magnitudes at the peak value were comparable. Based on the resulting similar trends, it was concluded that the simulation sequence was suitable to develop a new child seat.

The design of a six-point belt-type child seat was carried out resulting in a lightweight design to save material and manufacturing cost. However, such a lightweight design compromises the safety of the seat. Simulations varying the thickness of the material was carried out using ANSYS (A computer simulation software) to explore proper thickness vs safety tradeoffs. Results yielded a final design having 64.5% of its original volume. Once computer simulations were performed for the new six-point CRS, the sled test was carried out indicating that a six-point CRS provides a lower impact force due to the force being distributed over an increased area. (Park & Yoo 2009).

## Discussion

### Pediatric safety testing

#### Safety testing by anthropometric test devices (ATDs)

Children with disabilities often differ anatomically from children without disabilities, and therefore are often required to remain seated in their wheelchair while being transported in a motor vehicle. Injury risk of a child, seated in a manual pediatric wheelchair, was analyzed in this study using frontal impact sled testing. A 6HybridIII ATD was used, which models a non-disabled 6-year-old child with normal muscle tone and balance. Therefore, a child with disabilities may be more susceptible to severe and fatal injuries in circumstances where a child without disabilities would not be injured (Ha & Bertocci 2007).

This study showed that a 6-year-old seated in a wheelchair may be at risk of neck injury during a frontal car crash. The study also concluded that variation in the shoulder belt anchor point led to variance in restraint effectiveness. It was hypothesized that chest deflections would have been higher if the shoulder belt had been at a more optimum anchorage point (Ha & Bertocci 2007).

#### Safety testing by ATDs and computer simulations

Dynamic simulations of a child seat were carried out using LS-DYNA® to develop an advanced CRS design. Simulation results for a six-point belt-type child seat were compared to sled testing concluding that LS-DYNA® is a suitable alternative to replace sled testing, reducing cost and time for new product development (Park & Yoo 2009). However, it is acknowledged in this study that precise material properties are needed for accurate results.

## Conclusion

Studies have been carried out on the multiple aspects of toy-related injuries and the susceptibility of children with disabilities to injury. However, a gap in the literature occurs concerning the susceptibility of children with disabilities to toy-related injuries, specifically in relation to ride-on toys and the repercussions surrounding such injuries. It is theorized that such lack of data is due to the difficulty and costs associated with experimental validation. Hence, it is recommended that computer simulations be used to provide preliminary data analysis. Various aspects of small inertial impacts on a child with disabilities could be drawn from these studies. Furthermore, safety recommendations for ride-on toy modifications could be derived from such simulations and these could be correlated to specific disabilities. Ultimately the goal of such work would be to draw specific guidelines regarding modifications of ride-on toys and children with disabilities.

## Abbreviations

ATDs: Anthropometric test devices
CATIA™: Computer-aided three-dimensional interactive application
CDPF: The China Disabled Persons’ Federation
CRS: Child restraint system
FMVSS: Federal Motor Vehicle Safety Standards
HIC: Head injury criterion

## References

[CR1] Alemdaroğlu E, Öbudak SD, Mandiroğlu S, Biçer SA, Ã–zgirgin N, UÃ§an H. Predictive factors for inpatient falls among children with cerebral palsy. J Pediatr Nurs 2017;32:25–31. 10.1016/j.pedn.2016.08.005 27633845

[CR2] Blankenburg M, Junker J, Hirschfeld G, Michel E, Aksu F, Wager J (2018). Original article: quantitative sensory testing profiles in children, adolescents and young adults (6–20 years) with cerebral palsy: hints for a neuropathic genesis of pain syndromes. Eur J Paediatr Neurol.

[CR3] Gaw C, Abraham VM, Gaw CE, Chounthirath T, Smith GA (2015). Toy-related injuries among children treated in US emergency departments, 1990-2011. Clin Pediatr.

[CR4] Ginsburg KR (2007). Committee on communications, and committee on psychosocial aspects of child and family health. The importance of play in promoting healthy child development and maintaining strong parent-child bonds. Pediatrics.

[CR5] Ha DR, Bertocci G (2007). Injury risk of a 6-year-old wheelchair-seated occupant in a frontal motor vehicle impact—‘ANSI/RESNA WC-19’ sled testing analysis. Med Eng Phys.

[CR6] Kleinberger M, Sun E, Eppinger R, Kuppa S, Saul R. Development of Improved Injury Criteria for the Assessment of Advanced Automotive Restraint Systems. 1998 September,.

[CR7] Klevberg GL, Elkjaer S, Jahnsen R, Elvrum AG, Zucknick M, Ostensjo S (2018). Development of bimanual performance in young children with cerebral palsy. Dev Med Child Neurol.

[CR8] Lewandowski J. Federal Motor Vehicle Safety Standard (FMVSS) 208 – occupant crash protection: right front passenger test methodologies. Apr. 2006;3.

[CR9] Logan SW, Feldner HA, Galloway JC, Huang H (2016). Modified ride-on Car use by children with complex medical needs. Pediatr Phys Ther.

[CR10] Park D, Yoo W (2009). A study on the design of a child seat system with multipoint restraints to enhance safety. Journal of Mechanical Science & Technology.

[CR11] Rosenbaum P, Rosenbloom L (2012). Cerebral palsy : from diagnosis to adult life.

[CR12] Sinclair SA, Xiang H (2008). Injuries among US children with different types of disabilities. Am J Public Health.

[CR13] Thompson RA (2001). Development in the first years of life. Futur Child.

[CR14] Zhu H, Xiang H, Xia X, Yang X, Li D, Stallones L (2014). Unintentional injuries among Chinese children with different types and severity of disability. Ann Epidemiol.

